# Regulation of Endoplasmic Reticulum–Mitochondria Ca^2+^ Transfer and Its Importance for Anti-Cancer Therapies

**DOI:** 10.3389/fonc.2017.00180

**Published:** 2017-08-31

**Authors:** Gaia Pedriali, Alessandro Rimessi, Luigi Sbano, Carlotta Giorgi, Mariusz R. Wieckowski, Maurizio Previati, Paolo Pinton

**Affiliations:** ^1^Department of Morphology, Surgery and Experimental Medicine, Section of Pathology, Oncology and Experimental Biology, Laboratory for Technologies of Advanced Therapies (LTTA), University of Ferrara, Ferrara, Italy; ^2^Department of Biochemistry, Nencki Institute of Experimental Biology, Warsaw, Poland; ^3^Department of Morphology, Surgery and Experimental Medicine, Section of Human Anatomy and Histology, Laboratory for Technologies of Advanced Therapies (LTTA), University of Ferrara, Ferrara, Italy

**Keywords:** mitochondria-associated membranes, calcium, oncogenes, tumor suppressors, cell death, ROS, endoplasmic reticulum

## Abstract

Inter-organelle membrane contact sites are emerging as major sites for the regulation of intracellular Ca^2+^ concentration and distribution. Here, extracellular stimuli operate on a wide array of channels, pumps, and ion exchangers to redistribute intracellular Ca^2+^ among several compartments. The resulting highly defined spatial and temporal patterns of Ca^2+^ movement can be used to elicit specific cellular responses, including cell proliferation, migration, or death. Plasma membrane (PM) also can directly contact mitochondria and endoplasmic reticulum (ER) through caveolae, small invaginations of the PM that ensure inter-organelle contacts, and can contribute to the regulation of numerous cellular functions through scaffolding proteins such as caveolins. PM and ER organize specialized junctions. Here, many components of the receptor-dependent Ca^2+^ signals are clustered, including the ORAI1-stromal interaction molecule 1 complex. This complex constitutes a primary mechanism for Ca^2+^ entry into non-excitable cells, modulated by intracellular Ca^2+^. Several contact sites between the ER and mitochondria, termed mitochondria-associated membranes, show a very complex and specialized structure and host a wide number of proteins that regulate Ca^2+^ transfer. In this review, we summarize current knowledge of the particular action of several oncogenes and tumor suppressors at these specialized check points and analyze anti-cancer therapies that specifically target Ca^2+^ flow at the inter-organelle contacts to alter the metabolism and fate of the cancer cell.

## Introduction

From the 1940s, when a link between Calcium (Ca^2+^) and cancer was observed for the first time (1), until today, its centrality of Ca^2+^ action as second messenger in carcinogenesis and tumor progression has been confirmed (2).

Under resting conditions, the cytosolic Ca^2+^ amount is maintained at a concentration of approximately 100 nM. Some organelles act as intracellular Ca^2+^ stores, like the Golgi apparatus and endoplasmic reticulum (ER), and the concentration of this cation rises to between 300 and 1,000 µM in such places (3). Because of this high concentration gradient, modulation of intracellular Ca^2+^ homeostasis at the ER level is fundamental to cellular life and destiny. Rapid release of Ca^2+^ from the ER determines transient waves in the cytoplasm and mitochondria with pro-survival effects. On the contrary, stimuli that massively increase the mitochondrial Ca^2+^ concentration for a prolonged time induce apoptotic or necrotic cell death triggered by the opening of the mitochondrial permeability transition pore (mPTP) (3–5).

The mitochondria can accumulate a significant amount of Ca^2+^ within their matrix, 10-fold higher than that measured in the cytosol (6). Ca^2+^ is transferred from the ER *via* specialized regions, called mitochondria-associated membranes (MAMs) where the two organelles organize dynamic contacts (7). ER Ca^2+^ depletion, initiated by the opening of reticular inositol 1,4,5 trisphosphate (IP3) receptors (IP3Rs), is recovered by the sarco/endoplasmic reticulum Ca^2+^-ATPase (SERCA) pumps that transport Ca^2+^ into the lumen of the ER (8).

Increases in cytosolic Ca^2+^ concentration occur fundamentally through the entry of Ca^2+^ from the extracellular space. This event is mediated by ligand-gated channels, such as the P2X purinergic-ionotropic receptor families (9), and transient receptor potential (TRP) channels. As a whole, they constitute a superfamily organized into seven subfamilies, where one is comprised of the “canonical” TRPs (TRPC subfamily) (10). Moreover, some TRP channels can be influenced by the residual amount of Ca^2+^ in the ER after its release in the cytosol. Their action, which ultimately consists in the refilling the depleted stores, is termed store-operated Ca^2+^ entry (SOCE) and is regulated by the Ca^2+^ release-activated calcium channel protein 1 (ORAI1) and the ER Ca^2+^ sensors stromal interaction molecule 1 (STIM1) and STIM2 (11). STIM1 has been shown to redistribute into clusters or puncta at ER-plasma membrane (PM) junctional sites (12), while the caveolar lipid rafts form flask-like invaginations 50–100 nm deep in the cell. These structures, reducing the gap between the two membranes, facilitate SOCE channel interaction with ER-associated STIM1 puncta (13), and constitute a proper tether between the ER and the PM (13, 14).

Therefore, the control mechanisms of intracellular Ca^2+^ homeostasis appear hierarchical, and their modulation and alteration as a cause or consequence of cancer induction and progression can change the sensitivity of cells to anti-tumor drugs.

## Calcium and Cell Death

Ca^2+^ exerts a complex regulatory role on the numerous cell functions, including cell death (15). In particular, the overload of cellular Ca^2+^ is mediated in its pro-apoptotic signaling role, which also relies on the presence of a wide array of intracellular transducers and the high spatiotemporal complexity of the increase in [Ca^2+^] evoked by different apoptotic stimuli. Such complexity is controlled primarily by the presence of ion channels located in the PM and by structured inter-organelle interactions, such as between the ER and mitochondria (16). The importance of caveolae in Ca^2+^ signaling was confirmed by the strategic localization of Ca^2+^ effectors, such as PM Ca^2+^ ATPase pumps and IP3Rs, providing a platform for the assembly of diverse Ca^2+^ signaling complexes (17–20). In particular, the tumor-suppressor caveolin-1, a fundamental member of caveolae, plays a key role in the control of the Ca^2+^-dependent apoptotic pathway and regulates fundamental mitochondrial functions during tumor growth (21). When the caveolin-1/Ca^2+^ axis is compromised, failure of both mitochondrial metabolism, and apoptotic route can occur.

At ER-PM junctional sites, STIM-ORAI can sense and respond to intracellular Ca^2+^ microenvironmental changes; this complex mediates Ca^2+^ influx, while STIM acts as an ER Ca^2+^ sensor, ORAI serves as a selective Ca^2+^-entry channel. The over-activation of ORAI channels and TRPC result in Ca^2+^ toxicity caused by excessive Ca^2+^ influx (22). ORAI channels have a dominant role in Ca^2+^ toxicity because ORAI1 is essential for Ca^2+^ influx and regulates the activity of TRPC channels (23–25). The most potent and immediate regulator of ORAI1 is Ca^2+^ itself, with a pivotal contribution of STIM1 (26).

The luminal Ca^2+^ level controls IP3R-mediated Ca^2+^ release, dampening or augmenting ER-mitochondrial Ca^2+^ transfer, and consequentially shifting the balancing between cell death and survival. In particular, several anti-apoptotic proteins and oncogenes, such as bax inhibitor-1 (BI-1), B-cell CLL/lymphoma 2 (Bcl-2), AKT, and RAS reduce [Ca^2+^] in the ER lumen as a survival mechanism (21, 27–29). The activity of BI-1 as an ER Ca^2+^-leak channel and/or the sensitization of IP3Rs channels can driven the reduction of [Ca^2+^] at ER level, as demonstrated by the redox-related proteins ERO1α and GPX8 (30–32). In particular, the IP3R isoform 3 and voltage-dependent anion channel (VDAC)1 are proposed to have a powerful role in this pro-apoptotic Ca^2+^ signaling (33, 34).

Excessive Ca^2+^-release from the ER triggers mitochondrial pathways which can lead to cell death. Pro-apoptotic proteins [such as Bax and fragile histidine triad protein (Fhit)] exert the opposite effect, potentiating the mitochondrial Ca^2+^ signals, albeit by molecularly distinct routes: Bax antagonizes the effect of Bcl-2 on ER Ca^2+^ reload (35) while Fhit increases the number of the initial sites of Ca^2+^-uptake in mitochondria. Ca^2+^ overload in mitochondria has long been known to be a critical event in the metabolic impairment associated with both necrosis and intrinsic pathways of apoptosis (2, 21, 36–39). Ca^2+^ triggers the release of caspase cofactors such as cytochrome *c* and SMAC/direct IAP binding protein with low Pi, thus allowing the assembly of the apoptosome and driving the cell toward the opening of the mPTP, organelle fragmentation, swelling, and ultimately to death. Another important trigger for PTP opening is oxidative stress (40). Changes in the redox state impact ER and mitochondrial physiology and Ca^2+^ signaling. In particular, (i) the ER redox-related proteins, ERO1α, and GPX8, are enriched in MAMs and regulate ER Ca^2+^ storage and flux to mitochondria (30, 32) and (ii) the mitochondrial calcium uniporter (MCU) is a mitochondrial luminal redox sensor whose oxidation promotes persistent channel activity and Ca^2+^-overload-induced cell death (41–43). MCU provides the rate-limiting step for mitochondrial Ca^2+^ accumulation and may be pivotal to apoptosis (44). However, a large number of studies showed that MCU downregulation or inhibition increases resistance to apoptosis, in colon cancer cells *via* the upregulation of miR-25, a microRNA targeting the MCU itself (6).

## Oncogenes and Tumor Suppressors

**Figure 1:**
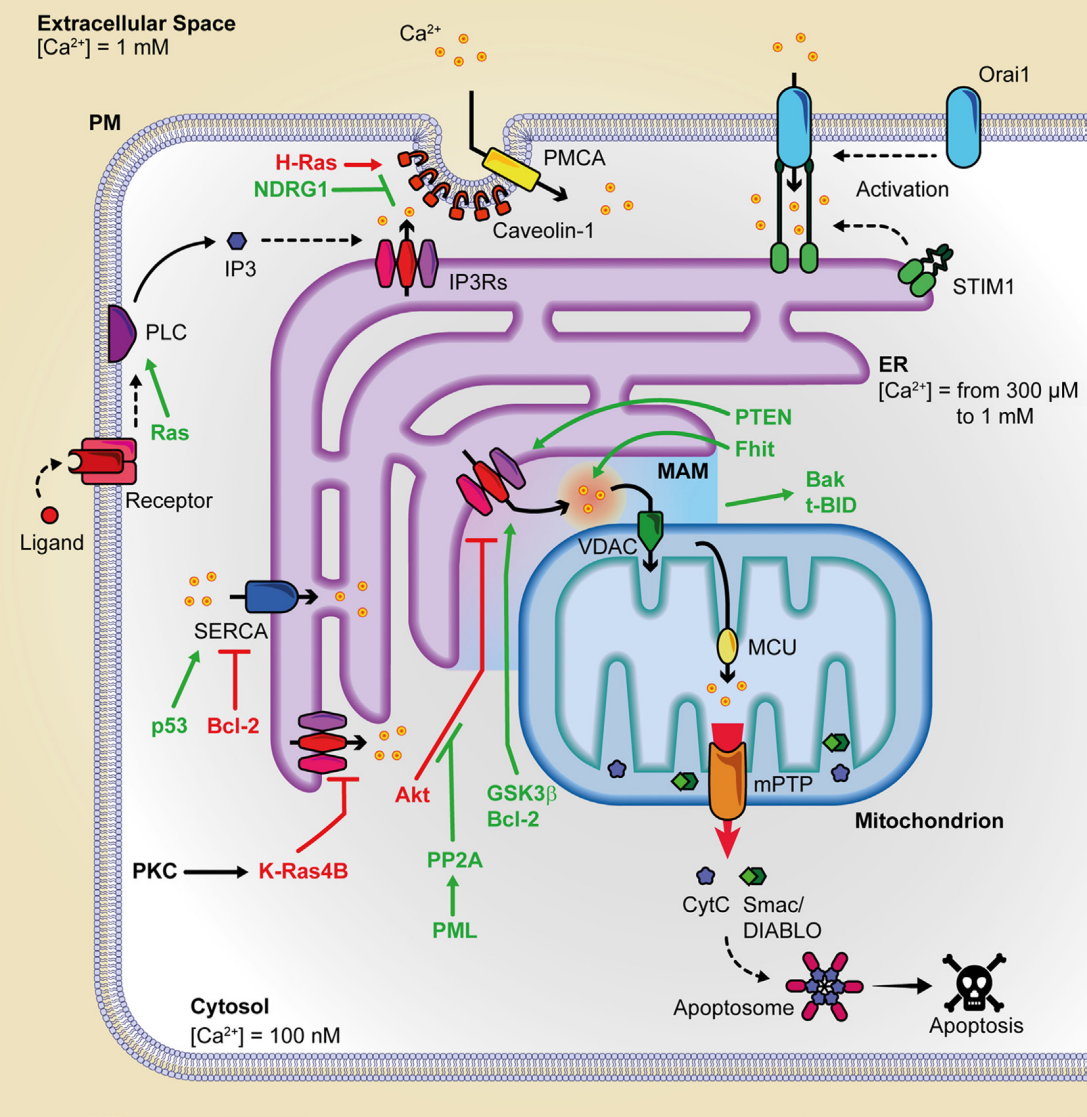
Summary of the principal oncogenes and oncosuppressors involved in inter-organelle Ca^2+^ transfer. This figure is a representation of the principal protein complexes involved in the intracellular Ca^2+^ transfer and of some of the various oncosuppressors and oncogenes that during cancer onset and progression can alter Ca^2+^ metabolism. Pro-apoptotic proteins are indicated in green, and anti-apoptotic proteins are depicted in purple. Bak, Bcl-2 antagonist/killer; Bcl-2, B-cell CLL/lymphoma 2; *c*; BID, BH3 interacting-domain death agonist; cyt. *c*, cytochrome *c*; ER, endoplasmic reticulum; Fhit, fragile histidine triad protein; GSK3, glycogen synthase kinase-3; IP3, inositol 1,4,5 trisphosphate; IP3Rs, inositol 1,4,5 trisphosphate receptors; MCU, mitochondrial calcium uniporter; mPTP, mitochondrial permeability transition pore; NDRG1, N-myc downregulated gene 1; PMCA, plasma membrane Ca^2+^ ATPase; PML, promyelocytic leukemia protein; PP2a, protein phosphatase 2PTEN, phosphatase, and tensin homolog deleted on chromosome 10; SERCA, sarco/endoplasmatic reticulum Ca^2+^ ATPase; SMAC/DIABLO, direct IAP binding protein with low Pi; VDAC, voltage-dependent anion channel.

Endoplasmic reticulum Ca^2+^ depletion plays a pivotal role in preventing mitochondrial Ca^2+^ overload and programmed cell death and, for this reason, it is the main target of the action of several oncogenes or oncosuppressors (Figure 1). In particular, STIM1 levels are upregulated in colorectal cancer and are positively correlated with tumor invasion and metastasis in this malignancy (45). STIM1 knockdown inhibited cell migration and invasion in both gastric cancer and glioblastoma (46, 47). ORAI1 and STIM1 regulate focal adhesion kinase at the front or at the rear edge of migrating cells, respectively (48). A front-to-rear Ca^2+^ gradient exists in these cells and is associated with STIM/ORAI-dependent Ca^2+^ pulses at the leading edge of the cell. This promotes myosin II-mediated directional movements and formation of new focal adhesions (49). Consistently, the use of the pharmacological SOCE inhibitor SKF-96365 attenuated tumor metastasis from MDA-MB-231 (45) or cervical cancer cell (50) xenografts. Also, the ORAI1/STIM1-dependent calcium entry in melanoma may drive the cell from a proliferative to a migratory and invasive state (51).

The PM protein Cav1 regulates tumor-associated cellular processes. Many studies have shown that Cav1 is a growth inhibitory protein, and its gene locus is often deleted in many cancers (52). Alternatively, its tumor-promoting activity and its augmented expression have been confirmed in a variety of cancers (53). Induction of oncogenic H-Ras leads to Cav1-mediated variations in intracellular Ca^2+^, associated with the alteration of mitochondrial physiology (21).

N-myc downstream-regulated gene 1 is a cytoplasmic protein deregulated in prostatic and colorectal cancers (54) that appears to behave as a metastatic suppressor. It interacts with and promotes the ubiquitylation of Cav1, therefore reducing its expression, depressing epithelial-to-mesenchymal transition, and weakening the metastatic capacity of colorectal cells *in vivo* (55). The pro-metastatic capacity of Cav1 is mediated by the S100 calcium-binding protein P (54) and is stimulated by hypoxic conditions, which increase Cav1 expression in hepatocellular carcinoma.

Reticular IP3Rs represent a main target of the action of oncogenes or oncosuppressors. In particular, IP3R3 is enriched in MAMs regions and appears to be the major player in the pro-apoptotic transfer of Ca^2+^ from the ER to mitochondria (33). The fact that IP3R3 is not inhibited by high concentrations of Ca^2+^ (56) implicates IP3R3, among its family members, as the principal effector of supramaximal pro-apoptotic mitochondrial calcium loading. Also, this suggests that other mechanisms must be involved in the control of IP3R3 receptor opening.

The oncogene Ras is a small GTPase mutated in a high percentage of human cancers, including pancreatic, colorectal, and lung cancers (57). Mutated and constitutively active Ras hyper-activates PLCε, with a consequent increase in IP3 and the downstream pathway. In addition to targeting IP3-producing enzymes, K-Ras has also been reported to remodel the expression of IP3R isoforms and SERCA2b at the ER level. Again, protein kinase C (PKC)-mediated phosphorylation of K-Ras4B induces its translocation to the ER where it can reduce cell survival by targeting IP3R3. Strikingly, mutated K-Ras suppressed mitochondrial Ca^2+^ dynamics in a Bcl-XL-dependent manner (58, 59). As such, mutated K-Ras may exert its pro-oncogenic role by dampening ER-originated Ca^2+^ release, so promoting malignant cell survival (60). Besides K-Ras, PKC isoforms contribute to the modulation of mitochondrial Ca^2+^ entry in response to a plethora to cell stimuli (61, 62).

Glycogen synthase kinase-3β (GSK3β) has been identified as a novel component of the MAMs, where it phosphorylates the IP3Rs and directly regulates them (63). In cardiomyocytes, ischemia reperfusion increased GSK3β activity, enhanced GSK3β-mediated IP3R phosphorylation and, in turn, mitochondrial-dependent cell death. Concerning VDACs, the exposure to apoptotic stimuli increases mitochondrial Ca^2+^ uptake through only the VDAC1 isoform (34), while VDAC2 acts on apoptosis in a Ca^2+^ independent manner triggering the mitochondrial recruitment of Bcl-2 antagonist/killer in tBH3 interacting-domain death agonist-induced apoptosis (64).

The proto-oncogene Bcl-2, discovered in the chromosomal translocation breakpoint t(14; 18) in B-cell follicular lymphomas (65), is upregulated in several cancers through chromosomal translocation, gene hypomethylation, and miRNA dysregulation mechanisms. Bcl-2 can interact with the IP3Rs through its N-terminal BH4 domain (66), regulating its channel properties (67), and sensitizing IP3Rs toward their agonist IP3. Also, Bcl-2 has been shown to join with SERCA1 and SERCA2b isoforms directly, consequently lowering their ER Ca^2+^-uptake activity (68, 69). As a whole, Bcl-2 contributes to the regulation of intracellular calcium stores by increasing the efflux of Ca^2+^ from the ER and thereby lowering the steady-state Ca^2+^-storage content at ER (70–72). The effect of Bcl-2 on ER Ca^2+^-level appears to be mediated by phosphorylation. PKA and JunN-terminal protein kinase (73) are among the kinases involved. Also, Bcl-2 targets the sixth transmembrane domain of the IP3R, contributing to inducing pro-survival Ca^2+^ oscillations.

Several reports have described the interaction of Akt with IP3Rs at the ER, resulting in the inhibition of Ca^2+^ release from IP3Rs after its phosphorylation by Akt, without affecting histamine-induced Ca^2+^ release or Ca^2+^ content (28, 74). Akt activity is balanced by PTEN, a tumor suppressor localized to the ER and MAMs that restores Ca^2+^ transfer from the ER to mitochondria. Its action does not depend upon lipid dephosphorylation, but upon the protein, dephosphorylation activity exerted directly on IP3Rs (75). It has been recently shown that PTEN can also counteract the binding of the F-box protein FBXL2 to IP3R3, where FBXL2 can target IP3R3 to proteasomal degradation so limiting the Ca^2+^ flux to mitochondria (76). Another potent tumor-suppresser gene that can exert part of its action by regulating Ca^2+^ flux to mitochondria is BRCA1-associated protein 1, which localizes to ER and binds, deubiquitylates, and stabilizes IP3R3 (77).

Also, promyelocytic leukemia protein (PML) participated to complexes with the protein kinase Akt and protein phosphatase 2a (PP2a), and its binding was critical for Akt- and PP2a-dependent downregulation of IP3Rs phosphorylation. In fact, the amounts of phosphorylated IP3R3 were higher in PML−/− than in PML^+^/^+^ MEFs cells, and higher levels of active phosphorylated Akt together with reduced amounts of protein phosphatase PP2a were found to be associated with IP3R3 inhibition (78). Consistently, the overexpression of PML made the cells sensitive to ER stress-induced apoptosis but not to calcium-independent cell death (78).

Recent studies demonstrated for cytosolic p53 at ER level a role in the regulation of protein interactions (79, 80). After chemotherapy, p53 accumulated at the ER and MAMs and reinforced H2O2-induced apoptosis; overall, it increased calcium accumulation in the ER and, consequently, in the mitochondria. p53 acted through a non-transcriptional mechanism, interacting through the C-terminal regulatory domain with the SERCA pump at the ER. Interestingly, wild-type p53 boosted Ca^2+^ accumulation and promoted apoptosis increasing SERCA activity, but oncogenic p53 mutants failed to stimulate it. p53 was reported to tether PML (81, 82). More specifically, Missiroli et al. (83), using PML−/− and p53−/− animal models, showed that p53 is indispensable to the recruitment of PML at MAMs.

## Anti-Cancer Therapies and Calcium

**Figure 2:**
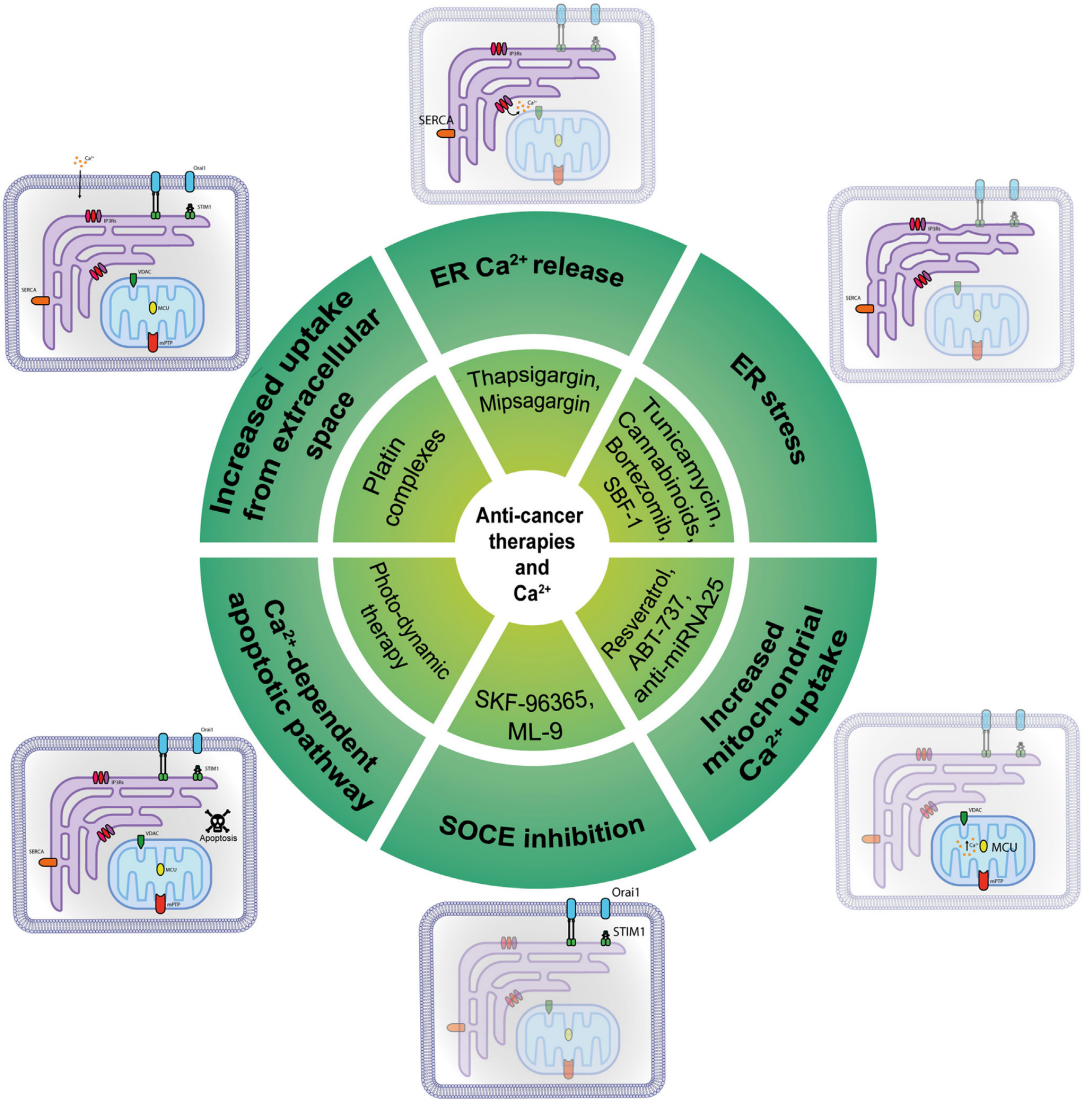
Anti-cancer molecules targeting intracellular Ca^2+^ transfer. Several classes of molecules can exert part of their anti-cancer action modulating at different levels the intracellular Ca^2+^ transfer.

Given the number of different Ca^2+^-transport mechanisms, therapeutic strategies have many potential targets to bring calcium homeostasis to normality in cancer cells and re-sensitize them to cell death and chemotherapeutic drugs (Figure 2). Initially believed to support the refilling of intracellular Ca^2+^ stores solely, SOCE has now been shown to sustain multiple calcium-dependent cancer pathways including uncontrolled cell proliferation; consequently, its pharmacological control is a primary target for cancer therapy. SKF-96365 is an ORAI1 inhibitor that can inhibit breast cancer cell migration *in vitro* and reduce tumor growth and metastasis *in vivo* (45); it also inhibited ORAI1-mediated SOCE and intracellular Ca^2+^ oscillations in esophageal cancer cells (84). On the other hand, it has been reported that SKF-96365 can activate autophagy, so delaying apoptosis in colorectal cancer cells *via* inhibition of the calcium/CaMKIIγ/AKT-mediated pathway (85). An inhibitor of SOCE is ML-9, through its interference with STIM1 (86). Although its target and mechanism of action are unclear, it was proved to disperse STIM1 puncta and to effectively induce prostate cancer cell death. Moreover, a combination of ML-9 and anti-cancer drugs, such as docetaxel, significantly promoted cancer cell death (87).

Drugs containing metal compounds can modify Ca^2+^ signaling and are commonly used to treat different types of tumors: platin complexes, such as cisplatin, carboplatin, and oxaliplatin, are used clinically to treat various types of cancers, including sarcomas, carcinomas, lymphomas, and germ cell tumors (88). For example, cisplatin causes increased uptake from the extracellular space, opening a membrane-associated calcium pore; this process involves membrane-associated IP3Rs. Hence, all of the compounds that can regulate Ca^2+^ could be considered a new class of chemotherapeutics, but their effectiveness could be insufficient when ER-mitochondria signal transmission is constitutively worsen, as in the case of Akt hyper-activation, or PML and PTEN inactivation. To overcome this obstacle, it might be useful to stimulate artificial ER Ca^2+^ release using the SERCA inhibitor thapsigargin. More specifically, conjugating thapsigargin to peptide substrates for prostate-specific antigen or prostate-specific membrane antigen (PSMA), it was possible to develope mipsagargin (G-202). G-202 is an inactive non-toxic prodrug that is activated only in PSMA-expressing epithelial cells, and in tumor vasculature, giving a high precision in tumor killing, specific to hit prostate, and other cancer cells (89). G-202 is in at the moment in the clinical phase of testing in several cancers including hepatocellular carcinoma (NCT01777594), prostate cancer (NCT02381236, NCT01734681), glioblastoma (NCT02067156, NCT02876003), and others. The only published results refer to a multicentre, open-label phase I study advanced, refractory, or metastatic solid tumors (NCT01056029), that reported an acceptable safety profile but no clinical response (90). The synthetic steroidal glycoside called SBF-1 causes severe ER stress by binding to and inhibiting SERCA2 activity, thereby causing cervical cancer cell death (91). In fact, ER stress might be used to obtain an anti-cancer effect: tunicamycin potentiates cisplatin anti-cancer efficacy, inducing accumulation of unfolded proteins in the ER (92), while cannabinoids activate the ER stress-related genes ATF-4 and TRB3, inducing pancreatic tumor cell death (93). Bortezomib (Velcade), a proteasome inhibitor recently approved for multiple myeloma, provokes ER stress in addition to requiring MCU as a critical regulatory factor in its activity (94). Velcade has been involved in more than 100 clinical testing with results. Together with its efficacy, several adverse events emerged, including thrombosis and embolism events, neuropathies, and other primary malignancies. The downside of these approaches is that tumor cells can use sustained ER stress to become more tumorigenic, metastatic, and drug-resistant and to escape to immune cells (95).

Another anti-cancer molecule is resveratrol, which selectively increases mitochondrial Ca^2+^ uptake in cancer cells after suppression of SERCA activity at the MAMs, while healthy cells remained unaffected (96, 97). Recently, a peptide based on the BH4 domain, which is the IP3R binding site of Bcl-2, can disrupt the interaction between these proteins and enhance Ca^2+^ release and consequent apoptosis (98). A modified peptide called BIRD-2 has recently been synthetized: it was found to provokes apoptosis in chronic lymphocytic leukemia cells (99) and diffuse large B-cell lymphoma cells (100). Multiple myeloma, follicular lymphoma, and small cell lung cancer cells also appear to be sensitive to BIRD-2 treatment (101, 102).

Another example of a BH3 mimetic involved in calcium remodeling is ABT-737, a non-selective Bcl-2/Bcl-XL inhibitor (103, 104); it can enhance ER-mitochondrial contact sites leading to Ca^2+^ overload at mitochondria and improving cisplatin’s toxic effect in human ovarian cancer cells (105). Recently, it has been tested in a trial concerning samples from ovarian cancers (NCT01440504), with no published results. A recent discovery recognizes miR-25 as a cancer-related MCU-targeting microRNA family that can be targeted with anti-miRNA 25 oligonucleotides; it could be used as a potential agent against cancer, as an alternative approach to hit tumor cells (6, 106).

## Conclusion

A growing number of findings indicate that several tumor suppressors and oncogenes can affect several levels of mechanisms regulating Ca^2+^ flow inside the cell, in addition to their well-known action on signal transduction pathways or on nuclear activities. The hierarchy of the Ca^2+^ transfer process involves several junctions in the highly compartmentalized cell interior, which warrant communication among different membrane systems. These junctions offer asylum to proteins that fill the role of oncogenes or tumor suppressors during cancer transformation, regulating the Ca^2+^ concentration inside the mitochondria and reticulum and consequently, altering cell metabolism, preventing apoptosis, and inducing cell migration. Specific therapies can target these junction complexes and revert Ca^2+^ flux to pro-apoptotic levels to sustain chemotherapy and other cancer therapies. These findings highlight the fundamental role of these inter-organelle junctions as hotspot domains, having pivotal, though not fully understood, roles in the regulation of cancer onset, and progression. Improving our knowledge of the regulation of Ca^2+^ transfer among these organelles will impact the search for new and more precise treatments for cancer.

## Author Contributions

Conception: GP, AR, MP, and PP. Design: GP, AR, LS, CG, MW, MP, and PP. Analysis and interpretation: GP, AR, LS, and CG. Drafting the manuscript for important intellectual content: GP, AR, LS, CG, MW, MP, and PP.

## Conflict of Interest Statement

The authors declare that the research was conducted in the absence of any commercial or financial relationships that could be construed as a potential conflict of interest.
